# *Drosophila* Kette coordinates myoblast junction dissolution and the ratio of Scar-to-WASp during myoblast fusion

**DOI:** 10.1242/jcs.175638

**Published:** 2016-09-15

**Authors:** Julia Hamp, Andreas Löwer, Christine Dottermusch-Heidel, Lothar Beck, Bernard Moussian, Matthias Flötenmeyer, Susanne-Filiz Önel

**Affiliations:** 1 Philipps-Universität Marburg, FB Biologie, Entwicklungsbiologie, Karl-von-Frisch Str. 8, Marburg 35043, Germany; 2 Fachbereich Biologie, Spezielle Zoologie, Philipps-Universität Marburg, Karl-von-Frisch Str. 8, Marburg 35043, Germany; 3 Interfaculty Institute for Cell Biology, Section Animal Genetics, University of Tübingen, Tübingen 72076, Germany; 4 Max Planck Institute for Developmental Biology, Section Electron Microscopy, Tübingen 72076, Germany

**Keywords:** Myogenesis, Myoblast fusion, WAVE, F-actin, Wip, Vrp1, Cellular junction

## Abstract

The fusion of founder cells and fusion-competent myoblasts (FCMs) is crucial for muscle formation in *Drosophila*. Characteristic events of myoblast fusion include the recognition and adhesion of myoblasts, and the formation of branched F-actin by the Arp2/3 complex at the site of cell–cell contact. At the ultrastructural level, these events are reflected by the appearance of finger-like protrusions and electron-dense plaques that appear prior to fusion. Severe defects in myoblast fusion are caused by the loss of Kette (a homolog of Nap1 and Hem-2, also known as NCKAP1 and NCKAP1L, respectively), a member of the regulatory complex formed by Scar or WAVE proteins (represented by the single protein, Scar, in flies). *kette* mutants form finger-like protrusions, but the electron-dense plaques are extended. Here, we show that the electron-dense plaques in wild-type and *kette* mutant myoblasts resemble other electron-dense structures that are known to function as cellular junctions. Furthermore, analysis of double mutants and attempts to rescue the *kette* mutant phenotype with *N-cadherin*, *wasp* and genes of members of the regulatory Scar complex revealed that Kette has two functions during myoblast fusion. First, Kette controls the dissolution of electron-dense plaques. Second, Kette controls the ratio of the Arp2/3 activators Scar and WASp in FCMs.

## Abstract (precis)

**Summary:** The *Drosophila* protein Kette is essential for myoblast fusion. It controls the dissolution of electron-dense plaques and the ratio of Scar and WASp proteins in fusion-competent myoblasts during fusion pore formation.

## INTRODUCTION

Myoblast fusion is fundamental for the formation of multinucleated muscles in mammals and *Drosophila*. The fusion of myoblasts requires many morphological changes in cells before the lipid bilayers of the plasma membranes mix and combine their cytoplasmic contents. In the first steps, myoblasts migrate towards each other, and recognize and adhere to each other. The plasma membranes of the adhering myoblasts are then destabilized, which leads to membrane fusion and to the formation of a growing muscle. The remodeling of cellular shapes depends on the reorganization of filamentous (F-)actin underneath the plasma membrane (Pollard and Borisy, 2003). In *Drosophila*, myoblast fusion is accompanied by modulation of F-actin at the site of myoblast contact (reviewed by Önel et al., 2011, 2014; Abmayr and Pavlath, 2012).

Specialized proteins, known as actin nucleators, mediate the formation of new actin filaments (Pollard, 2007). The most prominent actin nucleator is the evolutionarily conserved actin-related protein complex Arp2/3. The activity of this complex is also essential for induction of membrane fusion in myoblasts (reviewed by Önel, 2009; Gildor et al., 2010; Schejter and Baylies, 2010; Abmayr and Pavlath, 2012; Önel et al., 2014). Members of the Wiskott–Aldrich syndrome protein (WASp) family, and the Scar or WAVE proteins (Scar/WAVE; represented by the single protein, Scar, in flies) control the activity of the Arp2/3 complex (Rotty et al., 2013; Kurisu and Takenawa, 2009; Goley and Welch, 2006). WASp and Scar/WAVE proteins each have two common functional domains: the V domain, which binds to actin monomers, and the CA domain, which binds the Arp2/3 complex (Suetsugu, 2013), which together are referred to as the VCA domain. The binding of Arp2 and Arp3 subunits to the CA domain alters the conformation of both subunits and activates the complex (Robinson et al., 2001). The VCA domain is sufficient for Arp2/3 complex activation. The Scar/WAVE protein complex is inhibited by a pentameric regulatory protein complex that prevents the constant activation of the Arp2/3 complex. The *Drosophila* Nap-1 and Hem-2 (also known as NCKAP1 and NCKAP1L, respectively) homolog Kette is part of this regulatory complex. WASp is inhibited by an intra-molecular association of the WASp protein domains (Rohatgi et al., 1999; Kim et al., 2000; Derivery et al., 2009). Furthermore, WASp interacts with the WASp-interacting protein Wip (also known as Verprolin, Vrp1, and Solitary, Sltr, in *Drosophila*). Myoblast fusion in *vrp1* mutants is impaired (Massarwa et al., 2007; Kim et al., 2007; Berger et al., 2008).

In contrast to vertebrate genomes, *Drosophila* possesses only single *wasp* and *scar* genes, which contribute to different processes in development (Zallen et al., 2002). During somatic myoblast fusion, however, Scar and WASp are both essential for Arp2/3 activation (reviewed by Önel et al., 2014; Abmayr and Pavlath, 2012; Schejter and Baylies, 2010; Gildor et al., 2010). Myoblasts in *Drosophila* can be divided into two populations based on their molecular expression profile. Muscle founder cells determine the muscle identity (Bate, 1990) and fuse to fusion-competent myoblasts (FCMs). Upon fusion, the nucleus of the FCM adopts the identity and transcriptional profile of the founder cell, which is now referred to as a growing myotube (Baylies et al., 1998). Members of the immunoglobulin (Ig) and cadherin family are involved in recognition and adhesion of founder cells and FCMs (Bour et al., 2000; Ruiz-Gómez et al., 2000; Artero et al., 2001; Dworak et al., 2001; Strünkelnberg et al., 2001; Dottermusch-Heidel et al., 2012). However, only Ig-domain proteins are involved in the formation of a ring-like signaling complex (known as FuRMAS), which leads to Arp2/3-dependent F-actin formation at the cell–cell interface (Kesper et al., 2007; Richardson et al., 2007; Önel and Renkawitz-Pohl, 2009; Sens et al., 2010). Scar-dependent Arp2/3 activation in founder cells leads to the formation of a thin F-actin sheath (Sens et al., 2010). In FCMs, however, Scar and WASp cooperate to activate the Arp2/3 complex (Berger et al., 2008), which leads to the formation of a dense F-actin focus (Sens et al., 2010). The cytodomains of the Ig-domain proteins recruit cytoplasmic signaling proteins such as Nck (Kaipa et al., 2013) in FCMs, which serves as an adaptor protein for WASp and Scar complex members (Rivero-Lezcano et al., 1995). At the ultrastructural level, myoblast fusion is characterized by the appearance of electron-dense plaques, vesicles, actin-rich finger-like protrusions and fusion pore formation.

Although the allosteric regulation of Scar/WAVE and WASp has been studied extensively, little is known about how these multiple layers of regulation coordinate Arp2/3-dependent F-actin formation during organ formation, particularly during muscle formation. Recent research on myoblast fusion has focused on the formation of finger-like protrusions of FCMs that invade the founder cell or growing myotube (Sens et al., 2010; Kim et al., 2015). The formation of these finger-like protrusions seems to depend on WASp complex members (Jin et al., 2011) and not on Scar. What is then the function of Scar during myoblast fusion? In this study, we investigated the ultrastructural phenotype of *kette* mutants and showed that Kette is required for the dissolution of myoblast-specific cellular junctions containing N-cadherin. In contrast to the *kette* mutant phenotype, *scar vrp1* double mutants did not show extended cellular junctions. This finding indicated that Scar is required after myoblast-specific junction dissolution for the formation of a fusion pore. The ability of Scar to form a fusion pore was replaced by WASp in a *kette* mutant background. Our data further indicated that Kette coordinates the action of the Arp2/3 activators Scar and WASp by controlling the ratio of these proteins. From these data, we generated a model that highlights the different roles of Kette in branched F-actin formation during myoblast fusion.

## RESULTS

### Electron-dense plaques in wild-type and *kette* mutants are reminiscent of cellular junctions, and the removal of N-cadherin rescues the *kette* mutant phenotype

**Fig. 1.**
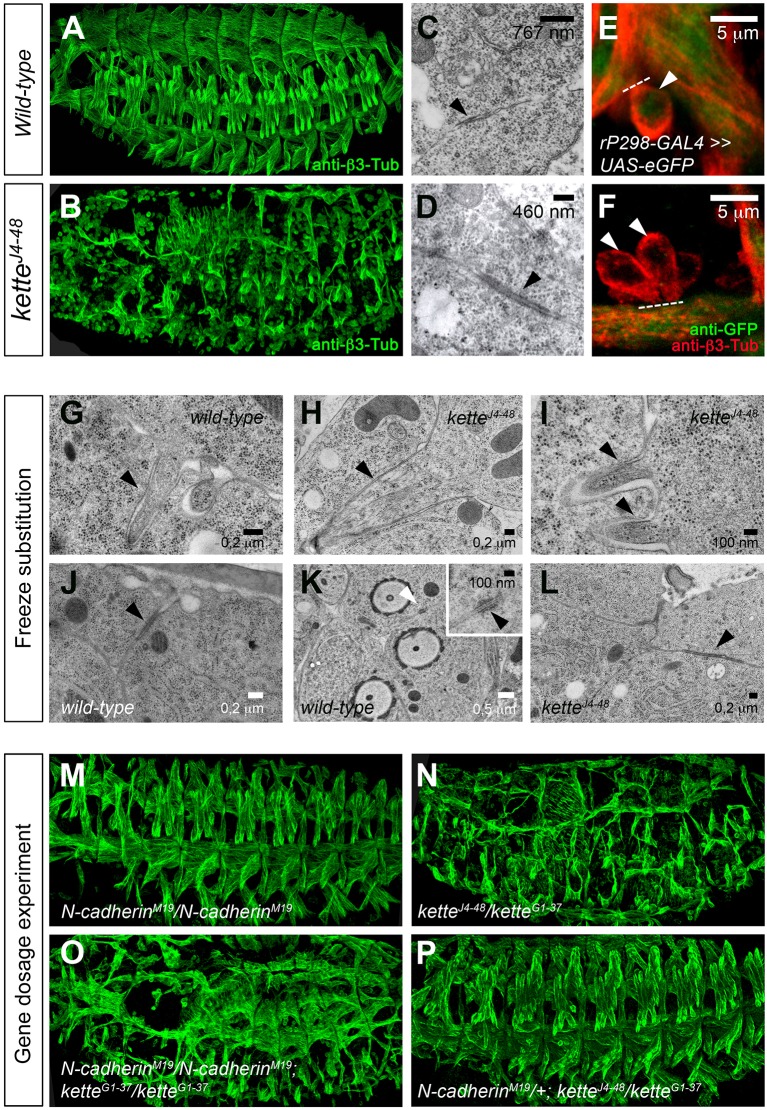
***kette* mutant myoblasts show aberrant cellular junctions and fail to form a fusion pore unless one copy of *N-cadherin* is removed.** (A,B,M–P) Lateral view of stage 16 embryos stained with anti-β3-Tubulin to mark all myoblasts and growing and mature muscles. (A) Wild-type. (B) Homozygous *kette^J4-48^* mutant embryo. (C,D) Transmission electron micrograph of stage 14 embryos conventionally chemically fixed. (C) Electron-dense plaque (arrowhead) between adhering myoblasts in a wild-type embryo. (D) Electron-dense plaque (arrowhead) between adhering *kette* mutant myoblasts. (E,F) GFP diffusion assay. Muscles of stage 15 embryo marked with β3-Tubulin (red) and expressing cytoplasmic GFP (green) in founder cells. (E) Diffusion of GFP from wild-type founder cell or growing myotube into the FCM (arrowhead). (F) No diffusion of GFP into the FCMs (arrowheads) from a homozygous *kette* mutant founder cell (growing myotube). (G–L) Transmission electron microscopy of stage 13 embryos using high-pressure freezing and freeze substitution. (G) Projection of a finger-like protrusion (arrowhead) from a wild-type FCM into a founder cell or growing myotube. F-actin filaments can be observed inside the protrusion. (H,I) Protrusions (arrowheads) containing F-actin filaments formed in *kette* mutant FCMs. (J) Adherens junctions between wild-type epithelial cells (arrowhead). (K) Septate junction (white arrowhead; black arrowhead in higher magnification) between tracheal cells during trachea development. (L) Electron-dense plaque (arrowhead) between *kette^J4-48^* mutant myoblasts. (M–P) Gene dosage experiments. (M) Homozygous *N-cadherin^M19^* null mutant embryo. (N) Transheterozygous *kette^J4-48^/kette^G1-37^* mutant embryo with severe myoblast fusion defects. (O) Dorsolateral view of a homozygous *N-cadherin^M19^; kette^G1-37^* mutant embryo showing the *kette^G1-37^* mutant phenotype. (P) Transheterozygous *kette^J4-48^/kette^G1-37^* mutant embryo lacking one copy of *N-cadherin^M19^*.

To investigate the role of Kette during electron-dense plaque formation, we reinvestigated the *kette* mutant phenotype using transmission electron microscopy (TEM) and a GFP fusion assay. Homozygous *kette* mutants carrying the *kette^J4-48^* null allele showed severe myoblast fusion defects (Fig. 1B; Table 1) compared to wild-type embryos (Fig. 1A, Table 1). We found electron-dense plaques of ∼500 nm in length in wild-type embryos (Fig. 1C) and electron-dense plaques that accumulated in *kette* mutants, as previously observed by Schröter et al. (2004) and Gildor et al. (2009) by using conventional chemical fixation. However, the length of the electron-dense plaques in *kette* mutants measured between 200 nm (Fig. S1E; Table S1) and 1 µm (Fig. 1D, arrowhead). Plaques that measured 1 µm in length were also found when we applied high-pressure freezing and freeze substitution to *kette* mutants (Fig. 1L, arrowhead; Table S1). The abnormal size of the plaque length indicates that *kette* function is associated with the plaques and that a fusion pore fails to form in *kette* mutants.

**Table 1.**
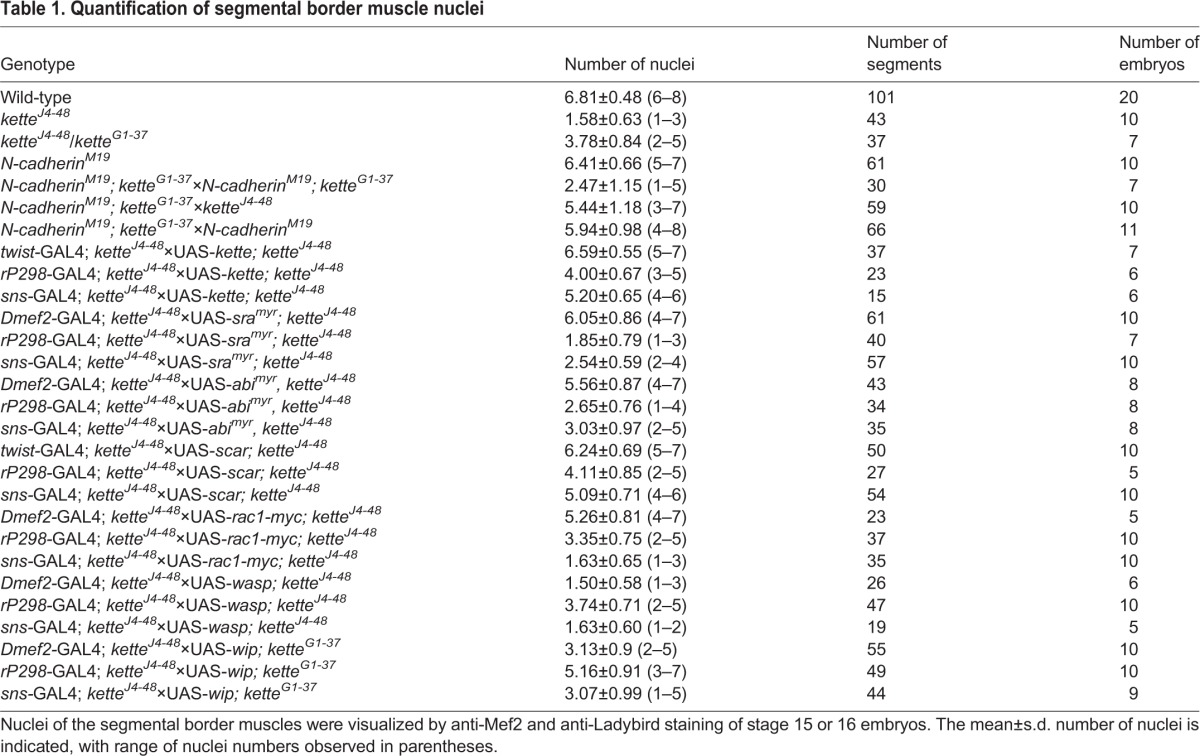
**Quantification of segmental border muscle nuclei**

We confirmed the inability of *kette* mutant myoblasts to form a fusion pore in a GFP diffusion assay. We observed that GFP was present in FCMs after specifically being expressed in founder cells, indicating that GFP had diffused into FCMs after fusion pore formation (Fig. 1E, arrowhead). However, GFP failed to diffuse into FCMs when expressed in *kette* mutant founder cells (Fig. 1F, arrowheads). Taken together, these results confirm that *kette* mutant myoblasts stop fusion prior to membrane breakdown.

The latest published data suggest that the formation of a fusion pore depends on the ability of myoblasts to form finger-like protrusions (Sens et al., 2010). By using conventional chemical fixation, we only detected finger-like protrusions once in a wild-type (Fig. S1B, arrowhead; Table S1) and in an *Arp3* mutant (Fig. S1C, arrowhead; Table S1). However, we observed fusion pore formation more often in *Arp3* mutants (Fig. S1C, arrows). For this reason, we used high-pressure freezing and freeze substitution to analyze whether *kette* mutant myoblasts are still able to form finger-like protrusions (Fig. 1G–I). We observed actin-rich protrusions in wild-type (Fig. 1G, arrowhead) and *kette^J4-48^* mutant myoblasts (Fig. 1H,I, arrowheads). From these data, we conclude that the failure of *kette* mutant myoblasts to fuse is due to the inability of electron-dense plaque dissolution and not due to being unable to form finger-like protrusions.

The function of the electron-dense plaques during myoblast fusion is still unclear. To elucidate their function, we first compared these structures (Fig. 1L, high-pressure freezing and freeze substitution; Fig. S1A,B, conventional chemical fixation) to two known cellular junctions, i.e. adherens junctions (Fig. 1J; Fig. S1G) and septate junctions (Fig. 1K), which also appear to be electron-dense at the ultrastructural level. Based on their ultrastructural similarity, we propose that the electron-dense plaques of myoblast fusion represent myoblast-specific cellular junctions. In the next step, we analyzed whether these plaques contain N-cadherin, because members of the cadherin superfamily are involved in the formation of cellular junctions, e.g. adherens junctions and desmosomes (Angst et al., 2001), and because we previously reported N-cadherin expression at the membrane of founder cells and FCMs (Dottermusch-Heidel et al., 2012). In gene dosage experiments in which we analyzed whether electron-dense plaques are N-cadherin-containing myoblast-specific cellular junctions that fail to dissolve in *kette* mutants, we removed one copy of *N-cadherin* in embryos that are transheterozygous for the *kette^J4-48^* null allele and the hypomorphic *kette^G1-37^* allele. Homozygous *N-cadherin^M19^* null mutants showed a wild-type-like muscle pattern (Fig. 1M, Table 1). Transheterozygous *kette^J4-48^/kette^G1-37^* (Fig. 1N) and homozygous *N-cadherin^M19^; kette^G1-37^* (Fig. 1O), however, displayed severe defects in myoblast fusion (Table 1). In addition, the ability of *kette^J4-48^/kette^G1-37^* mutant myoblasts to fuse was restored when one copy of *N-cadherin^M19^* was removed (Fig. 1P, Table 1). Furthermore, N-cadherin expression persisted longer in *kette^J4-48^/kette^G1-37^* mutant myoblasts or mini-muscles than in wild-type myoblasts (Fig. S1D–E′). Normally, N-cadherin expression is absent at stage 15 (Dottermusch-Heidel et al., 2012). These findings support the notion that the electron-dense plaques are N-cadherin-containing cellular junctions.

### Scar and Scar complex proteins seem to be required for cellular junction dissolution in *kette* mutants and are able to induce fusion pore formation

**Fig. 2.**
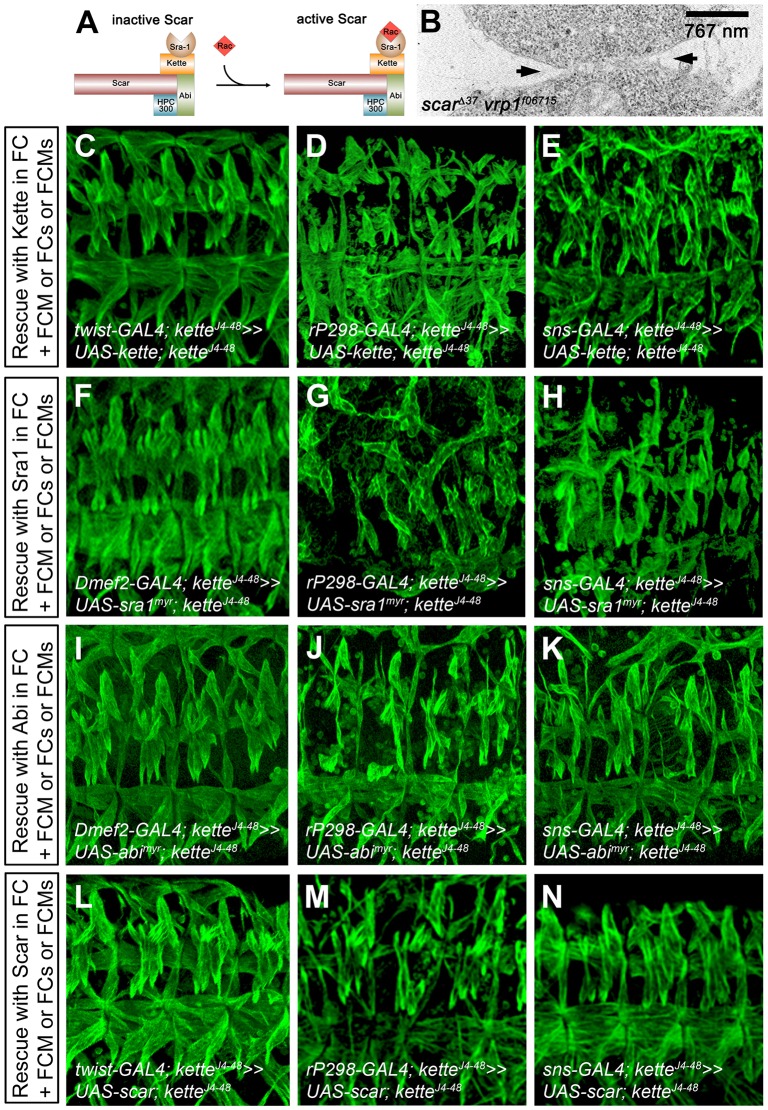
**Expression of Kette, Sra1, Abi and Scar in myoblasts rescues the *kette* mutant phenotype.** (A) Schematic representation of the Scar complex and its activation by Rac-GTPase. (B) Electron micrograph of a conventionally chemically fixed stage 14 *scar^Δ37^ vrp1^f06715^* mutant embryo. Membrane breakdown is visible (arrows). (C–N) Lateral view of stage 16 embryos stained with anti-β3-Tubulin. (C–E) Expression of UAS-*kette* (C) driven by *twist*-GAL4 in founder cells (FCs) and FCMs, (D) driven by *rP298*-GAL4 in founder cells and (E) driven by *sns*-GAL4 in FCMs, all in a homozygous *kette^J4-48^* mutant background. (F–H) Expression of myristoylated UAS-*sra1* (F) driven by *Dmef2*-GAL4 in founder cells and FCMs, (G) driven by *rP298*-GAL4 in founder cells and (H) driven by *sns*-GAL4 in FCMs, all in a homozygous *kette^J4-48^* mutant background. (I–K) Expression of myristoylated UAS-*abi* (I) driven by *Dmef2*-GAL4 in founder cells and FCMs, (J) driven by *rP298*-GAL4 in founder cells and (K) driven by *sns*-GAL4 in FCMs, all in a homozygous *kette^J4-48^* mutant background. (L–N) Expression of UAS-*scar* (L) driven by *twist*-GAL4 in founder cells and FCMs, (M) driven by *rP298*-GAL4 in founder cells and (N) driven by *sns*-GAL4 in FCMs.

Scar is essential in founder cells and FCMs for inducing Arp2/3-based F-actin polymerization (Sens et al., 2010). By contrast, WASp is only required in FCMs, where it cooperates with Scar to activate the Arp2/3 complex (Schäfer et al., 2007; Berger et al., 2008; Sens et al., 2010). Several groups have shown that WASp-dependent Arp2/3 activation is involved in the formation of a fusion pore (Massarwa et al., 2007; Berger et al., 2008; Sens et al., 2010; Jin et al., 2011). However, it is unclear whether Scar and Scar complex proteins (Fig. 2A) also contribute to fusion pore formation. Given that our data indicated that Kette is required for cellular junction dissolution in adhering myoblasts, we next asked whether Scar is also involved in this process. *scar* and the members of the Scar complex, *sra1* and *abi*, all possess a high maternal component. However, zygotic *abi* mutants or the expression of myristoylated, membrane-bound Sra1 (Sra1^Myr^) or Sra1ΔC^Myr^ that lacks the Kette interaction region (Bogdan et al., 2004) did not cause severe defects in myoblast fusion (Fig. S2C–E). Moreover, these genes are all required for oogenesis, and the induction of *scar*, *abi* and *sra1* maternal and zygotic germline clones leads to abnormal egg development (Hudson and Cooley, 2002; Zallen et al., 2002; Zobel and Bogdan, 2013). To avoid this problem, we analyzed *scar vrp1* double mutants by TEM; in these double mutants, myoblast fusion was stopped completely (Berger et al., 2008; Sens et al., 2010; Table 1). TEM of conventionally chemically fixed *vrp1* mutant embryos has revealed that *vrp1* mutants stop fusion during fusion pore induction (Massarwa et al., 2007); *vrp1* mutant embryos analyzed after high-pressure freezing and freeze substitution fail to form finger-like protrusions that are required for fusion pore formation (Sens et al., 2010). If Scar acts prior to Vrp1, we would expect to see adhering myoblasts with intact membranes and aberrant electron-dense plaques as in *kette* mutants. However, *scar^Δ37^ vrp^f06715^* mutants, like *vrp1^f06715^* mutants, stopped myoblast fusion during initiation of a fusion pore (Fig. 2B, arrows). We conclude from these results that Scar contributes to fusion pore formation after Kette-mediated cellular junction dissolution.

To gain further evidence that Scar and members of its associated complex are involved in fusion pore formation, we expressed Kette, Sra1, Abi or Scar in both founder cells and FCMs, or only in founder cells or FCMs in a *kette^J4-48^* mutant background and investigated their ability to rescue the *kette* mutant phenotype (Fig. 2C–N, Table 1). If Scar and members of its associated complex are only required for cellular junction dissolution, we expected to see no rescue of the *kette* mutant phenotype. However, we found that Kette, Sra1, Abi and Scar rescued the *kette* mutant phenotype when expressed in both myoblast types (Fig. 2C,F,I,L, Table 1). This finding suggests that the Scar complex is not only required for cellular junction dissolution, but also for fusion pore formation. We furthermore found that the expression of Kette or Scar only in founder cells or FCMs was sufficient to rescue the *kette* mutant phenotype (Fig. 2D,E,M,N, Table 1). However, Abi rescued the myoblast fusion defect of the *kette* mutant to a lesser extent than Kette or Scar when expressed only in founder cells or FCMs (Fig. 2J,K, Table 1). By contrast, expression of Sra1^Myr^ in founder cells failed to rescue the *kette* mutant phenotype (Fig. 2G, Table 1) and expression in FCMs only rescued the phenotype weakly (Fig. 2H, Table 1). Taken together, these data show that Kette, Sra1, Abi and Scar are required in both myoblast types. Moreover, these proteins are capable of inducing fusion pore formation when expressed in *kette* mutant myoblasts. This competence also applies to Kette or Scar when expressed in a specific myoblast type. However, the weak rescue by Abi or Sra1 when expressed in a specific myoblast type indicates that fusion pore formation mainly involves Kette and Scar.

### Expression of Rac1 in founder cells rescues the *kette* mutant phenotype and leads to a higher rescue when expressed in both myoblast types

**Fig. 3.**
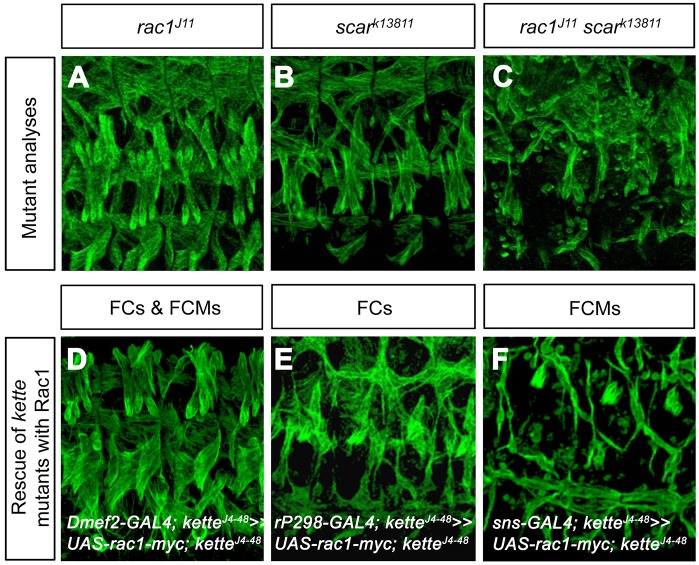
**Expression of Rac1 in founder cells, but not in FCMs, rescues the *kette* mutant phenotype.** (A–F) Lateral view of stage 16 embryos stained with anti-β3-Tubulin. (A) Homozygous *rac1^J11^* mutant embryo showing a wild-type muscle pattern. (B) Homozygous *scar^k13811^* mutant embryo showing a weak myoblast fusion phenotype. (C) Homozygous *rac1^J11^ scar^k13811^* double mutant embryo with severe defects in myoblast fusion. (D–F) Rescue of homozygous *kette^J4-48^* mutant embryos with Rac1 expression driven by (D) *Dmef2*-GAL4 in founder cells (FCs) and FCMs, (E) *rP298*-GAL4 in founder cells and (F) *sns*-GAL4 in FCMs.

Scar is activated by the binding of activated Rac to the Scar complex member Sra1 (Fig. 2A; Pollitt and Insall, 2009). The binding of Rac induces a conformational change of the Sra1–Kette subcomplex, which leads to the exposure of the VCA domain of Scar (Chen et al., 2010). During myoblast fusion, the *rac* genes *rac1* and *rac2* have overlapping functions (Hakeda-Suzuki et al., 2002). The loss of zygotic Rac1 or Scar did not induce myoblast fusion defects (Fig. 3A,B). By contrast, severe fusion defects were observed in *rac1 scar* double mutants (Fig. 3C). To determine whether the failure of Sra1 to rescue *kette* mutants when expressed in founder cells is due to a difference in Sra1–Kette subcomplex activation, we assessed the ability of Rac1 to rescue the *kette* mutant phenotype. We found that driving expression of Rac1 with *Dmef2*-GAL4 in both founder cells and FCMs rescued the *kette* mutant phenotype (Fig. 3D, Table 1). Similarly, the specific expression of Rac1 in founder cells with *rP298*-GAL4 enabled *kette* mutant myoblasts to fuse (Fig. 3E, Table 1). However, we observed no rescue when Rac1 was expressed only in FCMs in a *kette* mutant background (Fig. 3F, Table 1). Thus, we conclude that Rac1 is of particular importance in founder cells in a *kette* mutant background. However, this finding does not explain why the expression of Sra1 in founder cells fails to rescue the *kette* mutant phenotype.

### WASp and Vrp1 can replace Scar function in *kette* mutant founder cells, but not in *kette* mutant FCMs

**Fig. 4.**
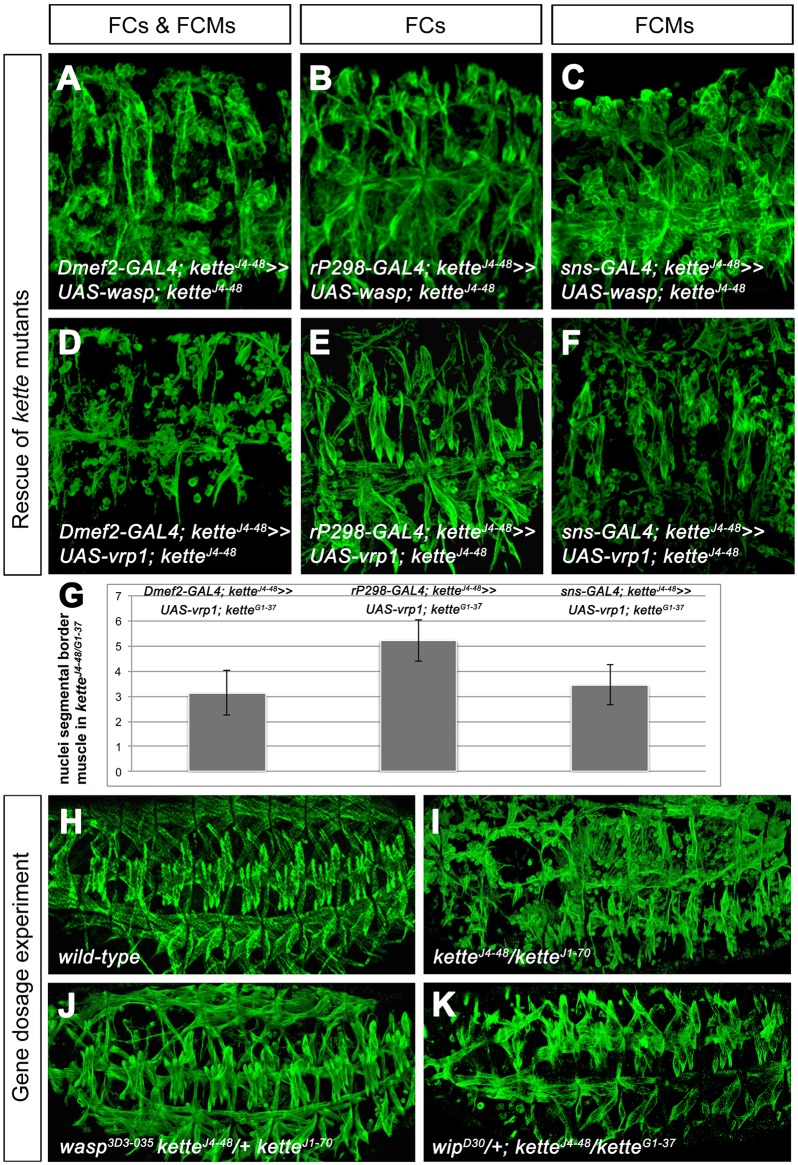
**Expression of WASp and Vrp1 in founder cells rescues the *kette* mutant phenotype, but also the removal of one copy of *wasp* or *vrp1*.** (A–K) Lateral view of stage 16 embryos stained with anti-β3-Tubulin. (A–C) Gene dosage experiments in which expression of UAS-*wasp* is driven by (A) *Dmef2*-GAL4 in founder cells (FCs) and FCMs, (B) *rP298*-GAL4 in founder cells and (C) *sns*-GAL4 in FCMs, all in a homozygous *kette^J4-48^* mutant background. (D–F) Expression of UAS-*vrp1* driven by (D) *Dmef2*-GAL4 in founder cells and FCMs, (E) *rP298*-GAL4 in founder cells and (F) *sns*-GAL4 in FCMs, all in a homozygous *kette^J4-48^* mutant background. (G) Quantification of fusions in transheterozygous *kette^J4-48^/kette^G1-37^* mutants in which the expression of UAS-*vrp1* was driven with *Dmef2*-, *rP298*- and *sns-GAL4*, as determined by the number of nuclei in the Ladybird-expressing muscle. Results are mean±s.e.m. for 10 embryos. (H–K) Gene dosage experiments. (H) Wild-type. (I) Transheterozygous *kette^J4-48^/kette^J1-70^* mutant embryos. (J) Removal of one copy of *wasp* in a *kette^J4-48^/kette^J1-70^* mutant background. (K) Removal of one copy of *vrp1* in *kette^J4-48^/kette^G1-37^* mutant background.

Up to this point, our data indicated that Kette is required for cellular junction dissolution in myoblasts and contributes, together with Scar, to fusion pore formation. The formation of a fusion pore also depends on the activity of WASp and its interaction partner Vrp1 in FCMs (Massarwa et al., 2007; Berger et al., 2008; Sens et al., 2010). Given that our data also point towards a function of Scar and WASp during fusion pore formation, we then asked whether WASp and Vrp1 can replace the function of Scar in founder cells. We again performed *kette* mutant rescue experiments, this time in which the expression of UAS-*wasp* and UAS-*vrp1* was driven in both myoblast types, only in founder cells and only in FCMs. Expression of WASp and Vrp1 driven by *Dmef2*-GAL4 in both myoblast types (Fig. 4A,D,G) or only in FCMs (Fig. 4C,F,G) failed to rescue the myoblast fusion defect of *kette* mutants (Table 1). However, the founder-cell-specific expression of WASp and Vrp1 induced myoblast fusion (Fig. 4B,E,G, Table 1). These data support the notion that WASp and Vrp1 are able to replace Scar function in founder cells and that Scar is required for fusion pore formation. However, these results do not explain why both Scar and WASp are needed in FCMs.

### Kette coordinates the ratio between Scar and WASp in FCMs

Recently, it has been reported that WASp dimerizes to activate the Arp2/3 complex and that this dimerization potentiates daughter nucleation (Padrick et al., 2011). In contrast to founder cells, FCMs are characterized by the formation of a dense F-actin focus during myoblast fusion. Thus, we speculated that the function of Scar and WASp in FCMs might be to potentiate branched F-actin polymerization. In *kette* mutants endogenous WASp is present in FCMs and we could show that the upregulation of Scar in *kette*-mutant FCMs is able to rescue the *kette* mutant fusion defect (Fig. 2N). This finding indicates that the ratio between Scar and WASp is essential to induce a fusion pore in *kette* mutants. To find further support for this notion, we reduced the wild-type gene dosage of *wasp* and *vrp1* in *kette* mutants. Transheterozygous *kette* embryos carrying the *kette^J4-48^* null and the *kette^J1-70^* hypomorphic allele showed severe defects in myoblast fusion (Fig. 4I, compare to wild-type in Fig. 4H). The removal of *wasp* in this genetic background rescued the *kette^J4-48^/kette^J1-70^* phenotype, i.e. muscle formation was restored (Fig. 4J). Next, we investigated whether the removal of *vrp1* also restored the ability of myoblasts to fuse in *kette* mutants. We removed one copy of the *vrp1* null allele named *wip^D30^* in the *kette^J4-48^/kette^G1-37^* mutant background. As expected, the *kette* myoblast fusion defect was repressed and muscle formation was restored (Fig. 4K). From these data, we conclude that Kette is an important coordinator of Scar and WASp function in FCMs and that the ratio between Scar and WASp is important to ensure myoblast fusion.

## DISCUSSION

**Fig. 5.**
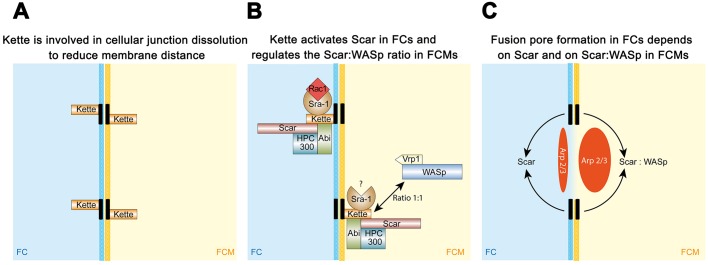
**Kette induces cellular junction dissolution and coordinates Scar- and WASp-dependent F-actin formation in FCMs.** Proposed model for Kette function during *Drosophila* myoblast fusion. (A) Kette is involved in the dissolution of a cellular junction-like structure that forms between adhering myoblasts and contains N-cadherin. The dissolution of the cellular-junction-like structures brings the membranes into close proximity for fusion. (B) Kette connects cellular junction dissolution with F-actin polymerization by recruiting members of the Scar complex to the site of fusion. Based on our genetic interaction studies, Rac1 might activate the Scar complex only in founder cells and not in FCMs. Our data further suggest that a specific ratio between Scar and WASp in FCMs is essential to promote fusion of myoblasts. (C) The resulting activation of the Arp2/3 complex in founder cells by Scar and in FCMs by a specific ratio of Scar and WASp initiates the formation of a fusion pore, which finally leads to myoblast fusion.

The ability of myoblasts to fuse depends on the precise regulation of Arp2/3-dependent F-actin polymerization. Scar/WAVE and WASp act differently in activating the Arp2/3 complex during myoblast fusion (Berger et al., 2008; Sens et al., 2010; Haralalka et al., 2011). Although many studies have addressed the function of WASp-dependent fusion pore formation during myoblast fusion at the ultrastructural level (Massarwa et al., 2007; Sens et al., 2010; Kim et al., 2015), not much is known about the role of Scar/WAVE during myoblast fusion. Our detailed analysis of *kette* mutants indicated that F-actin polymerization during myoblast fusion is more complex and does not only affect the finger-like protrusions and fusion pore formation. The multiple layers of Arp2/3 complex activation instead support a complex model, in which F-actin formation is required for myoblast-specific cellular junction dissolution as well as fusion pore formation in founder cells and FCMs. Fusion pore formation in FCMs, however, requires the precise coordination of Scar and WASp by Kette. To account for this, we modified existing models by adding these new findings (summarized in Fig. 5).

### Kette links myoblast-specific junctions with F-actin formation

In contrast to previous studies, we compared electron-dense plaques with electron-dense structures known to function as cellular junctions. Furthermore, we found that the removal of N-cadherin in *kette* mutants is essential for myoblast fusion to proceed. Based on these findings, we propose that the electron-dense plaques observed in *kette* mutants are N-cadherin-containing cellular junctions. The finding that the junctions are extended in *kette* mutants points to a function of Kette in the dissolution of these junctions (Fig. 5A). Kette is recruited to the membrane by the SH2-SH3 adaptor protein Dock, which is known as Nck in vertebrates (Kitamura et al., 1996). Recently, we demonstrated that Dock interacts with the Ig-domain proteins Sns, Hbs and Duf during myoblast fusion (Kaipa et al., 2013). Unlike N-cadherin, these proteins are expressed in a ring-like structure at myoblast contact points (Kesper et al., 2007; Sens et al., 2010; Dottermusch-Heidel et al., 2012). During fusion, this ring-like structure expands until the diameter of the myoblast is reached. This indicates that the Ig-domain proteins are shifted away from the site of contact during fusion. In contrast, N-cadherin is not expressed in a ring-like structure and is removed by a different mechanism from the site of contact that involves, as previously reported by Dottermusch-Heidel et al. (2012), the guanine nucleotide exchange factor Schizo (also known as Loner) and, as we found in this study, Kette. The presence of N-cadherin at the plasma membrane prevents the membranes being brought into close proximity for fusion. The binding of Dock to the Ig-domain proteins might recruit and activate Kette for dissolution of the N-cadherin-containing junctions. As a consequence, membranes are brought into close proximity and fuse. However, the loss of N-cadherin does not disturb *Drosophila* myoblast fusion. This is similar to mammalian myoblast fusion, where muscle (M-)cadherin seems to compensate for the loss of N-cadherin (Charlton et al., 1997). However, to date we have not identified a member of the cadherin family that can compensate for the loss of N-cadherin (Dottermusch-Heidel et al., 2012).

In TEM analyses of *scar vrp1* double mutants, we did not observe aberrant electron-dense plaques as in *kette* mutants. Instead, we found that *scar vrp1* mutants do not complete fusion pore initiation. Furthermore, expression of Scar in founder cells or FCMs rescued the *kette* mutant phenotype. Based on these findings, we propose that cellular junction dissolution occurs independently of Scar, and that Scar is required for the induction of a fusion pore in founder cells (Fig. 5C). This model is in accordance with studies on adherence junction formation in epidermoid carcinoma cells. In these cells, the Kette homolog Nap1, but not Scar/WAVE proteins, is involved in adherens junction formation (Ryu et al., 2009).

### Scar is required for fusion pore formation

The induction of a fusion pore in *scar vrp1* double mutants suggests that Scar functions in the absence of WASp during the first rounds of fusion to induce a fusion pore. The finding that the founder-cell-specific expression of Scar and WASp complex members rescues the *kette* mutant phenotype supports this notion. Recently, we found that the formation of multinucleated longitudinal visceral muscles depends only on the activity of Scar and not on the activity of WASp or Vrp1 (Rudolf et al., 2014). Longitudinal visceral muscles contain up to six nuclei per muscle, whereas somatic muscles contain four to 24 nuclei. Given that Scar is the only Arp2/3 regulator in longitudinal visceral founder cells and FCMs, membrane breakdown seems to depend only on Scar-based Arp2/3 activation in this context. During somatic muscle formation, the additional activity of WASp and Vrp1 is required afterwards, when the growing muscle further increases in size. This might explain why WASp and Vrp1 can replace Scar function in founder cells in a *kette* mutant background.

### Scar complex members modulate the stability and activity of Scar during myoblast fusion

Scar complex members control the stability of the Scar protein. The loss of any of these members leads to diminishing levels of the Scar protein (Kunda et al., 2003; Stradal and Scita, 2006; Takenawa and Suetsugu, 2007; Qurashi et al., 2007). In *Drosophila*, Abi, Kette, Sra1 and Scar are all maternally contributed and only zygotic *kette* mutants show a severe myoblast fusion phenotype. Between adhering *kette* mutant founder cells or growing myotubes and FCMs, less Scar protein is observed (Richardson et al., 2007). However, the rescue experiments in our study demonstrated that the observed diminished level of Scar protein can be rescued by upregulation of other Scar complex members in founder cells and FCMs, including Scar, in a *kette* mutant background. The myoblast-type-specific rescue of zygotic *kette* mutants also suggested that the regulation of the Scar complex in founder cells and FCMs might differ. Interestingly, we also found that upregulation of Rac1 rescues the fusion defect in zygotic *kette* mutants and thus positively modulates Scar stability. Myoblast-type-specific rescue experiments showed that this is only the case when Rac1 is upregulated in founder cells. This finding further supported the idea that the control of Scar stability in founder cells and FCMs differs.

### A specific ratio between Scar and WASp is essential for fusion pore induction in FCMs

The C-terminal VCA domain of the WASp protein family is mainly involved in stimulating the activity of the Arp2/3 complex (Takenawa and Suetsugu, 2007; Padrick and Rosen, 2010). The temporal and spatial activation of this domain is controlled by the N-terminal domains of the WASp protein family members (Burianek and Soderling, 2013). Besides this allosteric regulation of WASp family members in Arp2/3 activation (Miki and Takenawa, 1998; Rohatgi et al., 1999; Eden et al., 2002; Stovold et al., 2005), there might be an additional level of regulation by the dimerization of the VCA domain (Padrick et al., 2008). A model has been deduced from different studies in which the Arp2/3 complex *in vivo* has two VCA-binding sites (Padrick et al., 2008, 2011; Ti et al., 2011). However, other binding studies suggest a 1:1 ratio of the Arp2/3 complex and the VCA-binding site (Gaucher et al., 2012).

Studies on myoblast fusion have demonstrated that Scar and WASp are both required in somatic FCMs to induce Arp2/3-dependent F-actin foci formation, which is important to trigger membrane fusion. Thus, one challenge is to answer the question of how Scar and WASp become coordinated during myoblast fusion to activate the Arp2/3 complex. *kette* mutants fail to generate multinucleated muscles (Schröter et al., 2004) and have reduced levels of Scar (Richardson et al., 2007). We found that multinucleated muscle formation can be restored by reducing the *wasp* and *vrp1* gene dosage. This suggests that the ratio between Scar and WASp is important in FCMs for promoting myoblast fusion. Whether activation of the Arp2/3 complex in FCMs involves VCA dimerization needs to be clarified.

### Conclusions

Based on our results, we propose a new model for the function of Kette in cellular junction dissolution and fusion pore induction (Fig. 5). First, Kette links cell adhesion with F-actin formation and is thus important for the dissolution of myoblast-specific cellular junctions (Fig. 5A). Our genetic data indicated that these junctions contain N-cadherin. The cadherin extracellular region is 22 nm in length (Nagar et al., 1996). During indirect flight muscle formation it has been observed that the fusing myoblasts are brought into close apposition of less than 10 nm (Dhanyasi et al., 2015) before fusion pore formation. Thus, N-cadherin-containing junctions must be removed from the site of fusion to allow membranes to merge. Our TEM studies indicated that Scar acts after cellular junction dissolution. Second, Kette, Sra1, Abi, Rac1 and Scar are required for fusion pore formation in founder cells and FCMs (Fig. 5B). However, the functions of Sra1, Abi and Rac1 might differ in the two myoblast types. Moreover, gene dosage and myoblast-type-specific rescue experiments indicated that Kette coordinates the stoichiometric activity of Scar and WASp in FCMs (Fig. 5B). Thus, the activity of Scar in founder cells and the ratio of Scar and WASp in FCMs ensure the formation of a fusion pore between contacting myoblasts (Fig. 5C). Recent models suggest that fusion pore formation depends on the protrusive force generated by Arp2/3-based F-actin formation and by Myosin-II-dependent mechanical tension (Kim et al., 2015). Our study showed that Arp2/3-dependent F-actin formation is already required prior to fusion pore formation and that fusion pore formation depends on a precise balance of Scar and WASp function.

## MATERIALS AND METHODS

### *Drosophila* melanogaster lines and genetics

The *kette^J4-48^* and *kette^J1-70^* alleles were provided by Christian Klämbt (Münster University, Germany). The *N-cadherin^M19^* null mutant was provided by Tadashi Uemura (Kyoto University, Japan). UAS-*rac1*-myc was obtained from the Bloomington Stock Center. For the expression of UAS-transgenes, we used *sns4,5*-GAL4 (Stute et al., 2006), *snspro3*-GAL4 (Kocherlakota et al., 2008), *rP298*-GAL4 (Menon et al., 2001) and *Dmef2-*GAL4 (Ranganayakulu et al., 1996). Dmef2-GAL4 *kette^J4-48^*, UAS-*sra1-myr kette^J4-48^* and UAS-*abi-myr kette^J4-48^* fly strains were generated by meiotic recombination.

We used *Dr*/TM3 *Dfd-lacZ*, *If/*CyO *hg-lacZ* and *Sp*/CyO *wg-lacZ,* TM2/TM6 *ftz-lacZ* as blue balancers. All crosses were performed at 25°C using standard methods.

### Immunohistochemistry

Embryos were collected from grape-juice agar plates, dechorionated, devitellinized and fixed using standard methods. For each phenotypic analysis, at least 30 to 50 homozygous mutant embryos were analyzed by using a Leica TCS SP2 confocal microscope. The following primary antibodies were used: guinea pig anti-β3-Tubulin (1:10,000; Buttgereit et al., 1996; Leiss et al., 1988), rabbit anti-Dmef2 kindly provided by Hanh Nguyen (Erlangen University, Germany) (1:500), rabbit anti-Myc (1:2000, cat. no 05-724, Merck Millipore Darmstadt, Germany), rabbit anti-GFP (1:1000, ab6556, Abcam, Cambridge, UK), rat anti-N-cadherin DN-Ex#8 from Hybridoma Bank (1:500), and rabbit anti-β-galactosidase (1:5000, Cappel Research Products Durham, NC). As secondary antibodies, we used biotinylated antibodies from Vector Laboratories (Peterborough, UK) for DAB staining and Cy2- and Cy3-conjugated secondary antibodies from Dianova GmbH (Hamburg, Germany).

### Quantification of fusion

The fusion capacity of wild-type, *kette* null mutant, rescued *kette* mutant and double mutant embryos (Table 1) was analyzed by counting the nuclei of the segmental border muscle visualized with anti-Dmef2 and anti-Ladybird staining of stage 15 or 16 embryos. For each genotype analyzed, segmental border muscle nuclei of abdominal segments (A2–A7) of stage 15 or 16 embryos were counted.

### TEM analysis

We investigated wild-type embryos and *kette^J4-48^* mutants by using high-pressure freezing and freeze substitution, and by conventional chemical fixation and transmission electron microscopy (see Table S1). For conventional chemical fixation embryos were fixed as previously described in Berger et al. (2008). Ultrathin sections were obtained using an Ultracut E microtome (Reichert-Jung) and analyzed with a Hitachi HU-12A electron microscope. For high-pressure freezing and freeze substitution embryos were first dechorionated in bleach and then, without removing the vitelline membrane, cryo-immobilized by high-pressure freezing as described by Moussian et al. (2006). Samples were viewed in a Tecnai Spirit G2 electron microscope at 120 kV.
